# HEAL-Summ: a lightweight and ethical framework for accessible summarization of health information

**DOI:** 10.3389/fpubh.2025.1619274

**Published:** 2025-09-25

**Authors:** Andrew Fisher, Karthik Srinivasan, Sean Hillier, Vijay Mago

**Affiliations:** ^1^School of Health Policy and Management, Faculty of Health, York University, Toronto, ON, Canada; ^2^School of Business, The University of Kansas, Lawrence, KS, United States

**Keywords:** health communication, large language models, news summarization, semantic evaluation, information accessibility

## Abstract

**Introduction:**

The growing volume and complexity of health-related news presents significant barriers to public understanding. While large language models (LLMs) offer a promising means of summarizing such content, many approaches are computationally expensive and can lack sufficient evaluation of ethical as well as representational quality.

**Methods:**

To address these limitations, this research proposes a lightweight framework called Health Ethics & Accessibility with Lightweight Summarization (HEAL-Summ) for summarizing Canadian health news articles using LLMs. The framework incorporates three models (Phi 3, Qwen 2.5, and Llama 3.2) and integrates a multi-dimensional evaluation strategy to assess semantic consistency, readability, lexical diversity, emotional alignment, and toxicity.

**Results:**

Comparative analyses shows consistent semantic agreement across models, with Phi yielding more accessible summaries and Qwen producing greater emotional as well as lexical diversity. Statistical significance testing supports key differences in readability and emotional tone.

**Discussion:**

This work goes beyond single-model summarization by providing a structured and ethical framework for longitudinal news analysis, emphasizing low-resource deployment and built-in automated evaluations. The findings highlight the potential for lightweight LLMs to facilitate transparent and emotionally sensitive communication in public health, while maintaining a balance between linguistic expressiveness and ethical reliability. The proposed framework offers a scalable path forward for improving access to complex health information in resource-constrained or high-stakes environments.

## 1 Introduction

The accessibility of health information remains a challenge in public health, particularly in the context of sensitive issues such as mental health and the opioid crisis. Although news articles and health communication materials are widely available, their volume, variability, and linguistic complexity often make them inaccessible to the general public (1). This disconnect can contribute to misinformation, under-informed decision-making, and the widening of health disparities in underserved populations (2).

For example, in this study, over 30,000 health-related news articles were analyzed, with a mean article length exceeding 4,600 characters. Such information density highlights the barriers individuals may face when trying to interpret health communications at scale. Addressing this challenge requires tools that can condense, organize, and simplify complex information without sacrificing important content.

The challenge of information density is especially prevalent in the context of news articles, which serve as a primary information source for the public on issues such as mental health, substance use, and vaccination. While news media can play an important role in shaping health behaviors and perceptions, their linguistic complexity and variable framing may obscure essential content or contribute to misinformation (3). Automated summarization of health-related news offers a promising intervention to improve clarity, reduce cognitive burden, and promote informed decision-making (4).

Lightweight large language models (LLMs) offer a practical and efficient pathway for addressing this problem, enabling the generation of accessible summaries from large volumes of unstructured health-related text (5, 6). However, most existing approaches tend to rely on computationally intensive or costly models that are difficult to deploy in low-resource settings (7). For instance, foundation-scale LLMs such as GPT-4 Turbo operate exclusively through metered APIs that cost $0.01-$0.03 per 1,000 tokens for large-context usage, with additional completion charges.^1^ In contrast, recently released lightweight LLMs achieve competitive benchmark scores while running locally on consumer GPUs (8). Given the current cloud-GPU rates, one hour of local inference is significantly cheaper than the same volume of API-based inference, and on-device deployment removes network latency altogether.

To address these challenges, a lightweight LLM-based framework called HEAL-Summ (Health Ethics & Accessibility with Lightweight Summarization) is proposed using Phi 3, Qwen 2.5, and Llama 3.2 to summarize a dataset compiled from publicly available news articles in Canada (8–10). While many open-source LLMs are capable of generating summaries, existing implementations often focus on isolated, one-off summarization tasks. HEAL-Summ differs by offering a reproducible framework designed to support continuous, longitudinal summarization of health-related news while integrating important dimensions of ethical oversight. It evaluates model behavior not only by semantic similarity but also by accessibility, lexical expressiveness, emotional tone, and potential harms, which are factors that can impact public understanding and trust (11). Additionally, the system is optimized for low-cost, on-device inference, making it viable for use in settings where commercial API usage is impractical or unaffordable.

Focusing on topics of mental health, cancer, addiction, public health, vaccines, substance use, and suicide as case studies, the framework is applied to news media. As a result, the study makes the following contributions:

1. A novel framework, HEAL-Summ, for summarizing large-scale, real-world health content using low-resource LLMs suited for real-time applications.
2. A multi-faceted evaluation pipeline incorporating automated metrics to quantify readability, emotion, toxicity, and lexical diversity.
3. Model agreement and divergence analysis to assess semantic consistency and potential hallucinations across different LLMs.
4. Public release of LLM-generated news summaries with source URLs and open-source code for the summarization workflow, enabling transparency and reproducibility.

The remainder of this paper is organized as follows. In Section 2, related work on information processing and health communication is first reviewed, highlighting gaps in existing summarization and evaluation approaches. Next, a description of HEAL-Summ is presented, including data collection, filtering methods, and the LLMs used, as well as a multi-dimensional evaluation strategy. Section 3 reports the results across a dataset of publicly collected news articles. In Section 4, the implications, limitations, and future directions is discussed, then the article concludes with reflections on the role of responsible LLMs in public health.

## 2 Materials and methods

### 2.1 Materials

The integration of machine learning into healthcare and public-facing health communication has created new opportunities to transform large volumes of unstructured text into concise and comprehensible knowledge. Early advances such as BERTSum fine-tuned BERT for summarization tasks (12), while encoder-decoder architectures including BART and PEGASUS demonstrated state-of-the-art performance on news summarization (13, 14). In specialized domains, recent work has shown that LLMs can match or even surpass expert performance when generating clinical summaries of electronic health records (EHRs) (15).

However, many LLMs deployed in health applications remain computationally intensive or costly, limiting their viability in low-resource environments (7). Lightweight, open-source approaches have shown promise in related areas to address this concern, including question summarization (16) and text summarization tasks where smaller LLMs have been demonstrated to align closely with human-authored content (17). Extending these ideas to longitudinal health news coverage is particularly important, as media framing significantly influences public perceptions and behaviors concerning sensitive issues such as mental health, substance use, and vaccination (3).

Most summarization research, to the best of our knowledge, has focused on static inputs rather than continuously evolving information streams (18, 19). Timeline summarization (TLS) was an early effort to address this challenge by producing time-ordered summaries of events (20). Conventional TLS systems typically assume a homogeneous dataset centered on a single topic or query and output a unified timeline, often failing to capture multiple intersecting storylines (21). Other approaches, such as Multiple Timeline Summarization (MTLS), attempt to overcome this limitation by discovering distinct threads and generating timelines for each one (21). Nevertheless, these methods remain largely unsupervised (18), highlighting the need for scalable approaches that can accommodate large, diverse, and evolving corpora.

Another concern is ensuring that generated summaries are trustworthy, fair, and ethically aligned. Although LLMs can produce fluent and coherent text, they are prone to hallucination, meaning that they can introduce facts not present in the source material (22). Such inaccuracies can be particularly harmful in sensitive domains such as healthcare, as it can lead to the spread of misinformation (23). Furthermore, biases embedded within training data may lead models to generate distorted or unfair summaries, perpetuating societal stereotypes or toxic language (24, 25). Without appropriate safeguards, summarization systems risk reinforcing or amplifying harmful elements rather than mitigating them (26, 27).

Despite these challenges, gaps remain in evaluation and ethical oversight. Existing frameworks can lack integrated tools for real-time transparency, limiting their suitability for public health applications that directly impact vulnerable populations (28). Moreover, comprehensive evaluation remains limited, as beyond assessing informativeness, a useful summary should also preserve factual consistency, maintain readability, convey appropriate emotional tone, and avoid harmful language (29, 30).

To address these limitations, this study introduces HEAL-Summ, a lightweight summarization framework that applies low-resource LLMs to a large, evolving corpus of Canadian health news articles. The framework is designed to generate accessible, weekly summaries while embedding multi-dimensional evaluations of semantic consistency, readability, lexical diversity, emotional alignment, and toxicity. Unlike conventional systems that offer summarization as a discrete task, HEAL-Summ provides an integrated, end-to-end pipeline that supports ethical and longitudinal monitoring of public health discourse, optimized for local deployment in both high- and low-resource environments.

### 2.2 Methods

**Figure 1:**
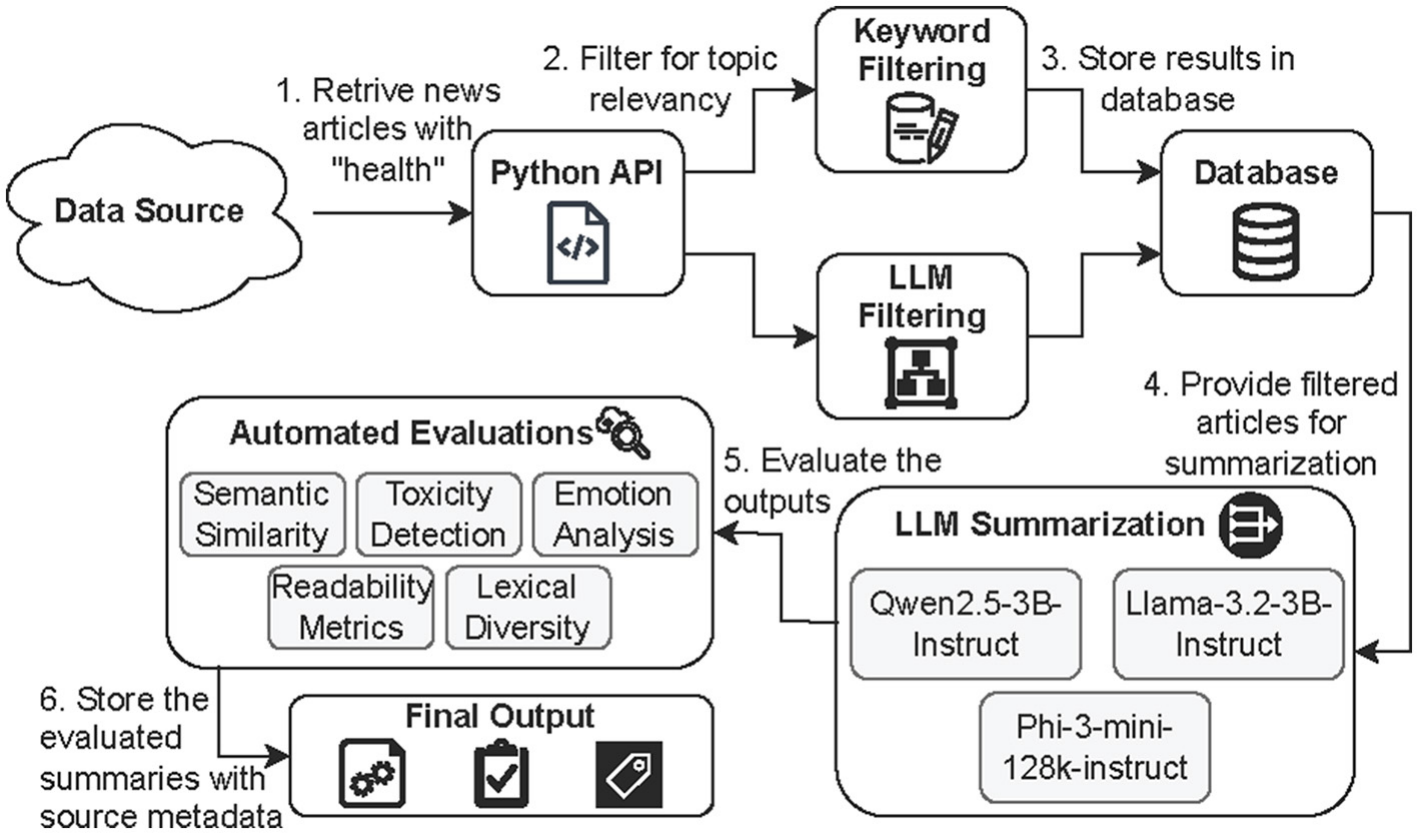
An overview of the proposed framework HEAL-Summ for health news summarization and evaluation. It supports real-time monitoring, flagging of potentially harmful content, and scalable deployment across diverse health topics.

HEAL-Summ is an automated pipeline designed for the real-time monitoring and summarization of public health discourse. As visualized in Figure 1, it comprises of the following stages: (1) automated retrieval of health-related news data, (2) topic filtering using both keyword-based and LLM-based methods, (3) summarization of filtered articles using multiple lightweight LLMs, (4) multi-dimensional evaluation of the generated summaries, and (5) storage of the evaluated outputs alongside source metadata. The framework supports longitudinal tracking of health-related narratives and enables efficient communication summaries. Its design accommodates both structured and unstructured inputs, and is focused on providing transparency, reproducibility, as well as flagging of potentially harmful content through automated evaluations.

#### 2.2.1 News article collection and preprocessing

In the demonstrated instance of the proposed framework, the MediaCloud API (31) is used for the data source to collect publicly available Canadian news articles from seven national and regional outlets: CBC News, City News, Globe and Mail, Global News, Thunder Bay News, The Star, and Winnipeg Free Press. These sources were selected based on their frequent appearance in returned queries from the API to provide a basis to demonstrate the framework. It does not fully reflect the diversity of the Canadian media landscape, but can be expanded using the comprehensive outlet collections that MediaCloud gathers information from Roberts et al. (31). Articles were first filtered using the keyword “health” and stored in structured JSON format. Each entry consists solely of text-based fields including the full text, title, URL, publication date, and outlet metadata. The final corpus consists of over 30,000 articles published in 2024 between January through October. Before downstream processing, all documents were stripped of HTML, lowercased, and tokenized.

The location of each article's JSON file was stored and managed using a local MySQL database, which served as a lightweight backend for subsequent filtering, summarization, and evaluation processes. Each article record retained its metadata and preprocessing status, with additional flags added to denote filtering decisions. This centralized storage approach enabled traceability between raw data, filtered inputs, and generated summaries, and supported temporal grouping as well as traceability across all stages of the framework.

#### 2.2.2 Topic filtering methods

To focus on topics such as “mental health” and “substance use,” two filtering strategies were applied to the weekly article sets. The first approach was static keyword filtering, where documents containing the topic's phrase were retained. While precise, this method may exclude semantically relevant articles that use alternate language or metaphors. To address this limitation, an LLM-based filtering approach was developed, where a lightweight model was prompted to read each article and assess whether it substantially covered the target topics by using the following prompt:

You are tasked with reviewing a POST and determining if it discusses TOPIC-related topics in Canada only. Provide YES or NO. Here is the POST:

This method allows for more flexible, context-aware filtering and enables a direct comparison between lexical filtering and adaptive semantic assessment. To further analyze unusual patterns or distributions in the filtered data, an additional unsupervised clustering step was applied to the article content for selected time windows of interest. This step was conducted manually after filtering, and was not used to determine relevance, but rather to explore the thematic composition of articles during specific periods. Article texts were first transformed into TF-IDF vectors to capture term relevance, and K-means clustering was used to group them based on semantic similarity, separate from the two filtering strategies (32, 33). Principal Component Analysis (PCA) was then used to project the resulting clusters for visualization (34). This post-filtering step enabled the identification of distinct thematic groupings within certain time periods, providing a structured view to explore the composition of news articles.

#### 2.2.3 Summarization framework

Filtered articles were organized by week of publication and grouped into weekly document sets. Each set was summarized using three lightweight LLMs: Phi-3-mini-128k-instruct (Phi, weights from July 2024), Qwen2.5-3B-Instruct (Qwen, weights from September 2024), and Llama-3.2-3B-Instruct (Llama, weights from September 2024) (8–10). These models were selected for their low computational requirements, extended context window capacities, and strong benchmark performance across instruction-following tasks. Furthermore, all three models support a context window of up to 128k tokens, allowing for large sets of articles to be processed at once. Summarization prompts were designed to extract concise, informative weekly summaries while preserving traceability to original sources. Model outputs were stored alongside structured metadata, including publication date ranges and URLs, enabling downstream evaluation of semantic consistency and readability.

#### 2.2.4 Evaluation strategy

To evaluate output quality of HEAL-Summ, a multi-dimensional evaluation pipeline is proposed. Each summary is assessed across semantic similarity to other models, emotions relative to the source articles, toxicity, readability, and lexical diversity. Because ground-truth reference summaries do not exist for the dataset, a relative and distributional evaluation strategy is adopted. This allows summaries to be compared against their source material as well as across models, enabling assessment of content consistency, language variation, and potentially harmful outputs without relying on predefined gold standards.

##### 2.2.4.1 Semantic similarity

To assess the degree of semantic consistency across models, cosine similarity was computed between summary embeddings using MiniLM-L6-v2, which is a transformer-based model (35). For each time period and filtering technique, summaries generated by the three LLMs were embedded using mean-pooling over token-level representations. Pairwise cosine similarity was then computed between these embeddings to quantify inter-model agreement. This evaluation captured how similarly the models interpreted and condensed the same input content. High similarity scores suggest semantic convergence and stable interpretation across models, whereas lower scores may reflect differences in the understanding of health content presented or potential hallucinations.

##### 2.2.4.2 Toxicity and emotion analysis

Toxicity was assessed using a RoBERTa-based toxicity classifier, which assigns a continuous toxicity probability to each input text (36, 37). Emotion were evaluated using the text2emotion library in Python,^2^ which outputs probability distributions across the following five classes: Happy, Angry, Sad, Surprise and Fear. Each source document is thus associated with a normalized score vector summing to 1.0, representing the relative presence of each emotion. To quantify emotional tone, the average predicted probability is calculated for each emotion category across all source articles and generated summaries. These aggregated scores enable a direct comparison of emotional content between the source texts and each model's outputs, where notable changes in the probabilities are interpreted as shifts in emotional framing. While emotion detection remains a proxy, this method offers a scalable way to monitor affective shifts introduced during summarization.

##### 2.2.4.3 Readability metrics

To assess the accessibility of the summaries, five standard readability indices were computed. These included the Flesch-Kincaid Grade Level (FK), Automated Readability Index (ARI), SMOG Index, Coleman-Liau Index (CLI), and Dale-Chall Readability Score (DC) (38, 39). Each of these metrics captures slightly different aspects of linguistic complexity. The FK estimates the U.S. school grade level required to understand a text, factoring in sentence length and syllables per word, using Equation 1:


(1) $$\begin{array}{l} FK=0.39\left(\frac{\text{words}}{\text{sentences}}\right)+11.8\left(\frac{\text{syllables}}{\text{words}}\right)-15.59 \end{array}$$

ARI similarly estimates readability but emphasizes character count and sentence length, as described in Equation 2:


(2) $$\begin{array}{l} ARI=4.71\left(\frac{\text{characters}}{\text{words}}\right)+0.5\left(\frac{\text{words}}{\text{sentences}}\right)-21.43 \end{array}$$

The SMOG Index predicts the number of years of education needed to comprehend a passage, particularly focusing on polysyllabic words, as per Equation 3:


(3) $$\begin{array}{l} SMOG=1.0430\sqrt{\text{number of polysyllabic words}\times \frac{30}{\text{sentences}}}\\ \;+3.1291 \end{array}$$

The CLI operates on characters per word and words per sentence, using the formula defined in Equation 4 designed for rapid digital computation:


(4) $$\begin{array}{l} CLI=0.0588L-0.296S-15.8 \end{array}$$

where *L*= the average number of letters per 100 words, and *S*= the average number of sentences per 100 words. Finally, DC compares the proportion of familiar words to a curated list of common vocabulary, identifying content that may be overly technical or dense as per Equation 5:


(5) $$\begin{array}{l} DC=0.1579\left(\frac{\text{difficult words}}{\text{words}}\times 100\right)+0.0496\left(\frac{\text{words}}{\text{sentences}}\right) \end{array}$$

where an additional 3.6365 is added if the percentage of difficult words exceeds 5% (39).

##### 2.2.4.4 Lexical diversity

To evaluate linguistic variation, lexical diversity was measured using the Measure of Textual Lexical Diversity (MTLD). Lexical diversity reflects how varied the word choices are within a given text and can offer insight into the models' ability to avoid repetition, over-reliance on generic phrasing, or excessive compression (40). This measure is especially relevant in public health, where clarity and precision are essential, but overuse of basic language may obscure nuances in the text or reduce reader engagement.

MTLD was selected over traditional type-token ratio (TTR) metrics due to its robustness to variations in text length. Unlike TTR, which typically declines as document length increases, MTLD calculates the mean length of sequential word strings in which the TTR remains above a given threshold (41). The result is a more stable indicator of lexical diversity across documents of differing sizes, such as summaries and their longer source texts.

##### 2.2.4.5 Ethical oversight and transparency

Ethical considerations are central to the design and implementation of HEAL-Summ. Inter-model divergence is used as a proxy for potential hallucination or instability (42). When semantic similarity between models diverges significantly for the same input, it may suggest inconsistent grounding in the source content. This triangulation-based approach aligns with techniques such as SelfCheckGPT (43), which use ensemble disagreement to flag factual inconsistencies. Combined with emotion and toxicity analyses, this method provides a scalable proxy for ethical evaluation in the absence of large-scale manual annotation.

While full-scale human evaluation was not feasible, periodic manual reviews were conducted throughout the development cycle. These reviews served as qualitative sanity checks to validate the coherence, factuality, and tone of summaries (29). Cases of inconsistent or overly generic outputs informed refinements to the prompt templates and evaluation thresholds. To promote transparency and reproducibility, all summaries generated from the news dataset are released alongside the original article URLs, as well as the code used to analyze the results.^3^ Although full-text redistribution of news articles is restricted due to copyright considerations, the inclusion of direct links supports traceability and allows end-users to verify summarized content (44).

## 3 Results

HEAL-Summ is evaluated across several dimensions to assess the quality, accessibility, and ethical suitability of model-generated summaries. These include semantic consistency, readability, lexical diversity, and emotion alignment. All evaluation metrics are computed over weekly summaries, with results stratified by model. Tables that include an “Average” column reflect the mean value across the three LLMs (i.e., Qwen, Llama, and Phi).

### 3.1 Model agreement and semantic similarity

To assess the degree of semantic consistency across models, the cosine similarity between summaries generated from the same weekly article set was computed using MiniLM-L6-v2 (35). While the overall average similarity across all topics was approximately 0.779, separating the results by health topic in Table 1 reveals some differences in model behavior. On high-level issues such as vaccines and substance use, similarity scores were high across all models with averages exceeding 0.828, suggesting that the LLMs tend to converge on common messaging when article content is focused or drawn from centralized public health guidance.

**Table 1:** The average inter-model similarity across weekly summaries for each health topic.

**Topic**	**Qwen**	**Llama**	**Phi**	**Average**
Vaccine	0.843	0.844	0.828	0.838
Substance use	0.835	0.833	0.816	0.828
Addiction	0.795	0.801	0.785	0.794
Public health	0.777	0.774	0.761	0.771
Suicide	0.757	0.802	0.749	0.769
Mental health	0.738	0.746	0.725	0.736
Cancer	0.735	0.734	0.700	0.723

Similarity is computed using cosine distance between sentence embeddings of summaries generated by each model on the same article batch, with the overall average across all models presented in the last column.

In contrast, mental health topics for example exhibited more variability. While the average similarities remained between 0.725 and 0.746, the values were overall lower, suggesting that linguistic ambiguity or narrative framing differences in these source articles may affect how different models prioritize content. This may be due to variations in tone, coverage styles, or terminology involving emotional states. A similar result could be observed for cancer, which had the lowest average similarity among the topics analyzed, with scores around 0.700–0.735. This may reflect the differences of articles under this label, ranging from survivorship stories to policy coverage or clinical trial updates, leading to more divergent interpretations across models.

### 3.2 Readability analysis

**Figure 2:**
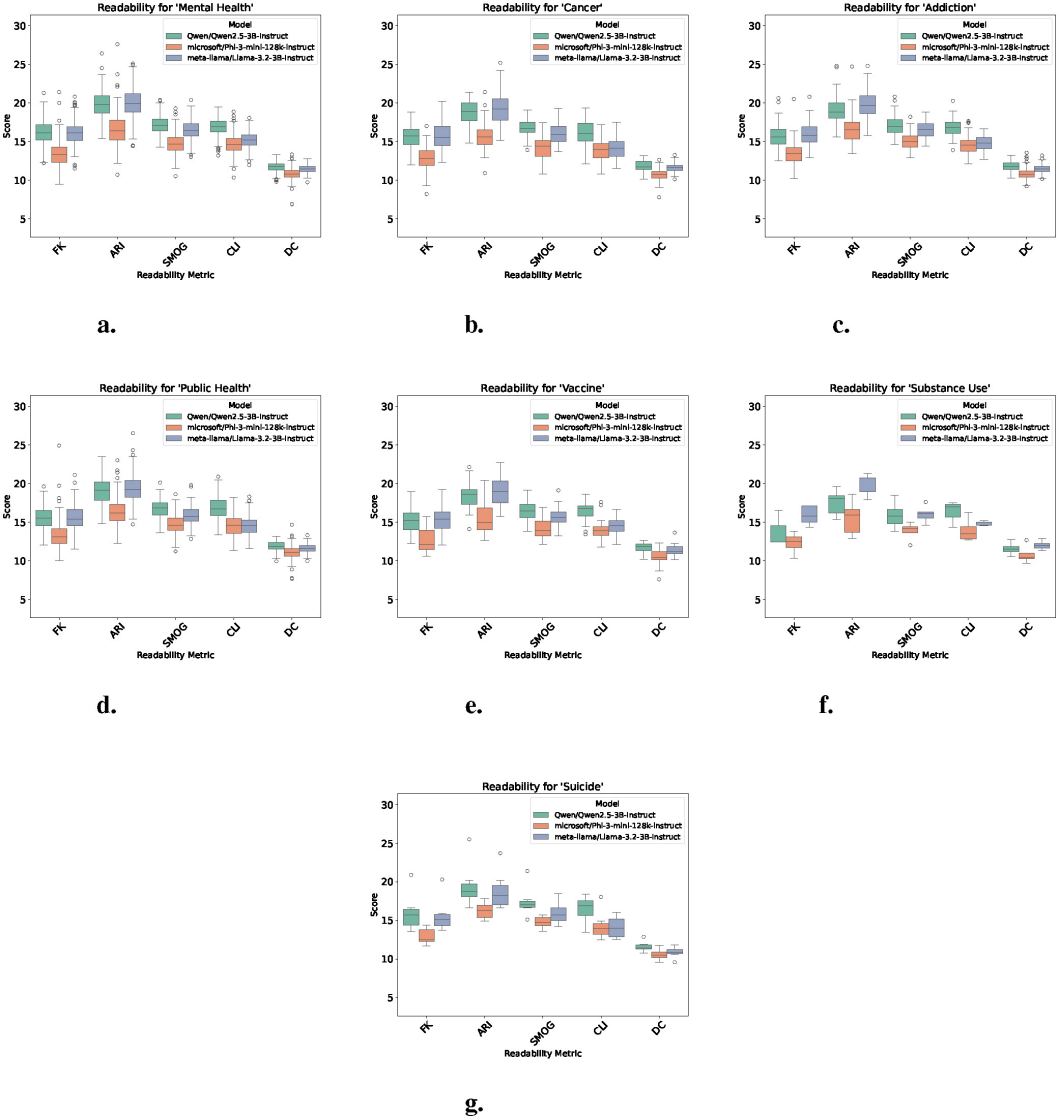
Readability scores across models for each topic. Each plot shows five readability metrics per model. Lower values generally indicate more readable summaries. **(a)** Readability scores for “mental health.” **(b)** Readability scores for “cancer.” **(c)** Readability scores for “addiction.” **(d)** Readability scores for “public health.” **(e)** Readability scores for “vaccine.” **(f)** Readability scores for “substance use.” **(g)** Readability scores for “suicide.”

To evaluate summary accessibility, five standard metrics were computed. Demonstrated in Figure 2, across all topics (Figures 2a–g), Phi consistently generated the most readable outputs with lower scores on nearly every index. This trend was particularly evident in topics such as public health (Figure 2d), vaccine (Figure 2e), and substance use (Figure 2f), where Phi's output metrics remained lower than those of Qwen or Llama.

In contrast, Qwen and Llama produced less readable summaries, often reflecting higher grade levels and greater variability. For instance, in mental health (Figure 2a) and addiction (Figure 2c), both models showed elevated FKGL scores that occasionally exceeded a score of 20, while Phi remained lower and more readable. In suicide (Figure 2g), readability differences between models narrowed, but Phi still trended lower. This was consistent across nearly all summaries generated as Phi maintained lower and more consistent readability scores over time, while Qwen and Llama fluctuated more. These patterns suggest that, based on readability scores, Phi may be comparatively more suitable for generating simplified, public-facing summaries. However, models Qwen and Llama may offer advantages in lexical complexity or stylistic expressiveness, depending on the context and communication goals.

### 3.3 Lexical diversity

Lexical diversity, measured by MTLD, highlights clear stylistic differences across models and topics as shown in Table 2. Qwen consistently demonstrates the most diverse vocabulary, with the highest mean MTLD scores in every topic. This trend is especially evident in topics such as cancer and public health, where the metric shows that Qwen's outputs span a broader lexical range. In contrast, Phi produces the least complex language, reflected in its consistently lower MTLD values, while Llama typically falls in the middle. These findings are similar to the readability results, indicating that models with greater lexical diversity often generate less readable summaries.

**Table 2:** The average Measure of Textual Lexical Diversity (MTLD) across models and health topics.

**Topic**	**Qwen**	**Llama**	**Phi**	**Average**
Cancer	197.25	153.80	139.43	163.49
Public health	186.72	151.66	143.72	160.70
Addiction	178.96	146.36	140.65	155.32
Vaccine	175.15	140.45	135.46	150.35
Substance use	175.81	144.10	129.82	149.91
Mental health	168.97	137.70	133.92	146.86
Suicide	169.38	141.18	128.42	146.33
Average	178.89	145.04	135.92	

Higher values indicate greater lexical variation. The overall average of these scores across all models is presented in the last column.

### 3.4 Emotion alignment

**Figure 3:**
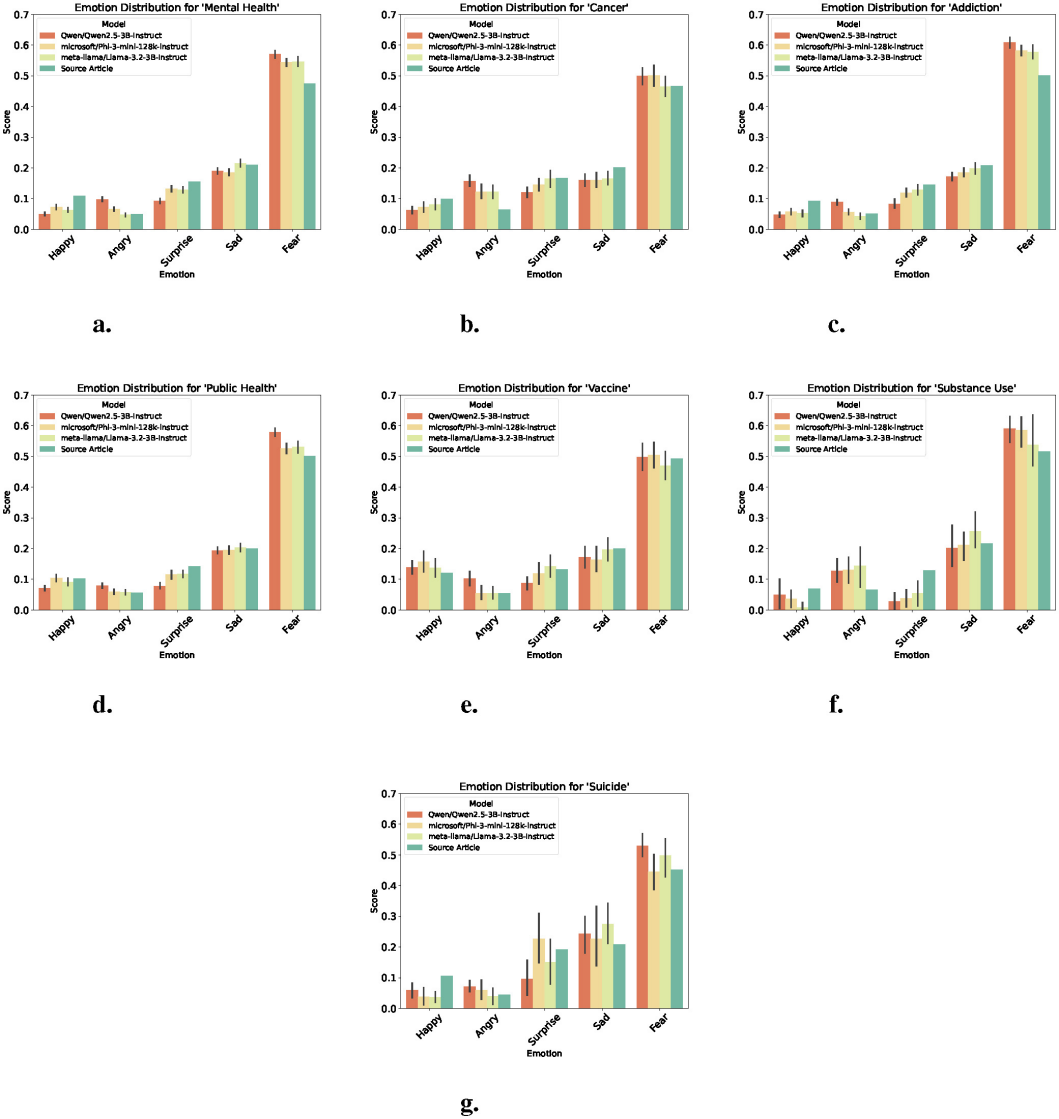
Emotion scores across models and source articles by topic. Each plot shows the average expression of five core emotions. Higher values indicate more prevalence of the emotion in the text. **(a)** Emotion scores for “mental health.” **(b)** Emotion scores for “cancer.” **(c)** Emotion scores for “addiction.” **(d)** Emotion scores for “public health.” **(e)** Emotion scores for “vaccine.” **(f)** Emotion scores for “substance use.” **(g)** Emotion scores for “suicide.”

Emotion detection was used to assess how well each model preserved or altered the emotional tone of the original news articles. Using probability vectors, the average emotion score across all documents for each category were computed. As shown in Figure 3, all summaries generally reflect the dominant emotional cues found in the source texts, with fear consistently prominent across all categories (Figures 3a–g). However, differences can be observed across models and topics, such as Qwen which diverged from the emotional profile of the source more frequently, especially in more sensitive topics such as cancer (Figure 3b) and addiction (Figure 3c). Phi periodically softens negative emotions such as anger, while slightly amplifying positive emotions such as happy, notably in topics such as suicide (Figure 3g) and vaccine (Figure 3e) respectively. Llama generally follows source trends, but shows a tendency to proportionally match the trends of other models, possibly contributing to a more neutral or softened emotional tone.

**Figure 4:**
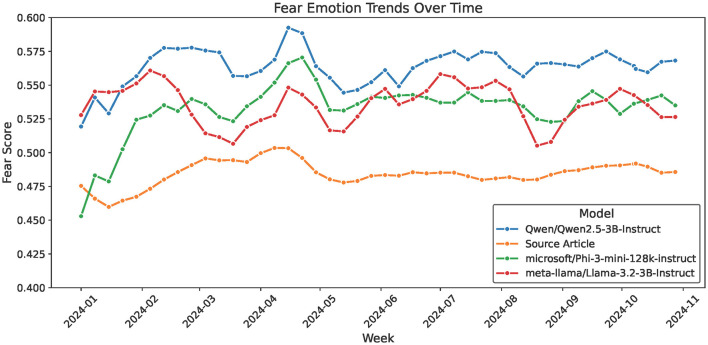
A temporal view of a rolling average in the trend of fear-related emotion scores across all health topics and source articles.

These variations may suggest that each model exhibits a unique pattern of emotional shaping, which could reflect differing optimization goals for safety, neutrality, or affective calibration. Despite these nuances, core emotional signals are broadly preserved, confirming the models' alignment with the thematic tone of health-related news content. Given that fear emerged as the most dominant emotion across all topics, a follow-up analysis was conducted to observe how this emotion evolved over time across models and in comparison to the source articles. As shown in Figure 4, while all models increase the overarching fear-driven tone of health news, differences in temporal sensitivity can be observed. Qwen more significantly increases the week-to-week trajectory of fear in source content, whereas Phi and Llama falls in between, preserving some variation.

**Figure 5:**
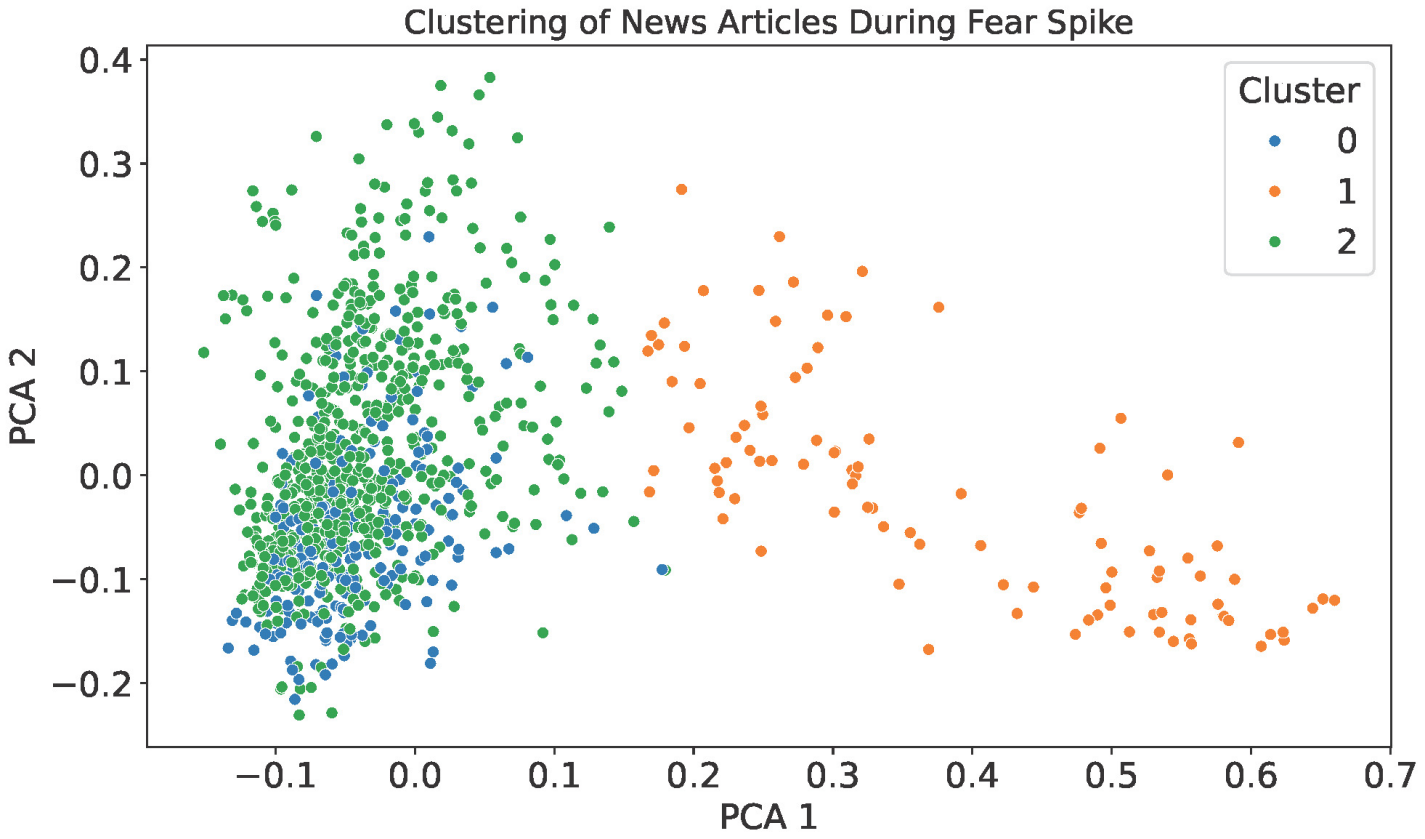
A 2D projection of news articles published during the April–May 2024 fear spike observed in Figure 4, clustered using TF-IDF and K-means (*k* = 3). Each point represents an article, and colors indicate cluster membership.

In all instances, it can be observed that there is a spike of fear between April (2024–04) and May (2024–05). To better understand this increase, the filtered articles from this period were clustered using TF-IDF and K-means, as shown in Figure 5. The resulting three clusters capture related but distinguishable thematic concentrations after examining the article contents. Cluster 1 is centered around drug policy and public safety, with frequent mentions of terms such as “decriminalization,” “overdose,” and “opioid.” Clusters 0 and 2 exhibits an overlap, as both contain discussions related to mental health, healthcare services, and public well-being. However, they differ in their dominant emphasis, as Cluster 0 focuses primarily on youth mental health and education, featuring terms such as “school,” “students,” and “children,” whereas Cluster 2 captures broader healthcare concerns, such as cancer care, hospital services, and community health programs. Although thematic boundaries between these clusters are not perfectly distinct, separating them offers interpretive value by highlighting the different social domains through which fear was expressed during this period. The clustering analysis thus demonstrates the complementary role of emotion tracking and unsupervised exploration in contextualizing shifts in public health discourse.

### 3.5 Toxicity evaluation

Toxicity was assessed using a RoBERTa-based model, which assigns a probability of toxic content on a 0–1 scale (36, 37). Across all models and topics, toxicity scores remained near zero, with the largest observed difference between summaries and their corresponding source articles being less than 0.0011. This held true even in sensitive topics such as addiction and mental health, suggesting that the models compiled summaries without introducing or amplifying overly harmful language.

Given the consistently low toxicity levels, a comparative plot is not included as it would offer limited interpretive value. The most “toxic” summaries, which had scores no higher than 0.01, were consistently observed to be associated with sensitive subject matter, including suicide, sexual exploitation, and medical assistance in dying. These summaries did not exhibit toxic language, but rather the slight elevation in this metric's score may stem from the emotional tone or traumatic nature of the underlying topics.

### 3.6 Statistical significance

To further explore differences between models beyond aggregate scores, pairwise *post-hoc* significance tests were conducted to control for multiple comparisons. The results, as shown in the Supplementary material, indicate that several evaluation metrics yielded statistically significant differences between specific model pairs. For MTLD, all model pairs demonstrated significant differences, with Qwen producing more lexically diverse outputs than Phi and Llama (adjusted *p* = 0.000), and Phi also differing significantly from Llama (adjusted *p* = 0.002). As discussed in the results, this reinforces the finding that Qwen tends to generate more varied vocabulary in its summaries.

In terms of readability, Phi was found to produce significantly more accessible outputs across all five metrics. For the FK, Phi differed significantly from both Qwen and Llama (adjusted *p* = 0.000), as previously noted where Phi consistently yielded lower scores. ARI confirmed this pattern, with Phi again differing significantly from both Qwen and Llama (adjusted *p* = 0.000), and even Qwen and Llama showing divergence (adjusted *p* = 0.001). Similar trends were evident across CLI and DC, where Phi produced significantly more readable outputs than the other models (adjusted *p* = 0.000). SMOG further confirmed this, with all pairwise comparisons between Phi reaching significance (adjusted *p* = 0.000). Together, these results validate Phi's observed tendency toward simpler sentence structures and vocabulary, making it particularly suitable for public-facing summaries, as discussed earlier.

Several emotional tone categories also showed meaningful divergence. For fear, Qwen differed significantly from both Llama and Phi (adjusted *p* = 0.000), suggesting stronger or more explicit framing in its summaries. For anger, all pairwise comparisons were significant, with Qwen expressing higher levels overall. For happiness, Qwen again differed from both models (adjusted *p* ≤ 0.001), whereas Phi and Llama were more aligned (adjusted *p* = 0.122). Lastly, Qwen also diverged significantly from Llama in its expression of sadness (adjusted *p* = 0.000), and from both Llama and Phi in its handling of surprise (adjusted *p* = 0.000). These findings highlight the importance of model choice when deploying summarization systems in emotionally sensitive contexts, especially in domains such as public health communication where tone and accessibility directly impact comprehension and trust.

### 3.7 Qualitative comparison

To complement the quantitative evaluation, a qualitative comparison of model outputs was provided to illustrate how differences in model behavior manifest in tone, structure, and accessibility. Examples are presented in the Supplementary material across three dimensions: readability, lexical diversity, and semantic similarity. For readability, samples were selected based on average scores across the five metrics, highlighting both more and less readable summaries for each model. More readable outputs tended to be concise, clearly segmented, and focused on a narrow set of developments. For instance, one of Phi's most readable summaries centered on focused narratives, such as updates related to Canadian public figures and cancer diagnoses, providing short, declarative sentences with minimal syntactic complexity. In contrast, less readable outputs from Qwen and LLaMA were lexically dense, incorporating multiple storylines or regions.

Lexical diversity was assessed through examples with high and low MTLD scores. Summaries with lower diversity often repeated similar terminology or framed content in highly standardized ways. For instance, Qwen's lower-diversity summary emphasized recurring themes such as mental health and opioid policies with similar vocabulary. In contrast, high-MTLD summaries, demonstrated richer language variation, bringing together nuanced discussions of substance use, mental health, public safety, and health policy.

Finally, semantic similarity across model outputs was qualitatively assessed by comparing summaries generated for the same article sets. The high-similarity examples showed consistent thematic extraction, centered on singular public health developments such as vaccination updates. Conversely, low-similarity examples revealed differences in focus and detail prioritization across models, such as divergent emphases on public figures' health diagnoses or national health trends. These cases may suggest that while models generally agree on major themes, stylistic and interpretive variability can emerge in more complex or multi-faceted topics. To support practical model selection, Table 3 summarizes the relative strengths, limitations, and suggested deployment contexts for each model, based on the observed quantitative and qualitative results.

**Table 3:** Summary of model characteristics based on evaluation results and recommended contexts for use.

**Model**	**Pros**	**Cons**	**Recommended use**
Qwen	High lexical diversity; emotionally expressive; preserves nuanced tone shifts	Lower readability; amplifies fear and anger	Contexts requiring rich detail or expressive tone (e.g., policy briefs, technical summaries)
Llama	Balanced tone and moderate complexity; emotionally stable	Mid-range readability and lexical diversity; less distinctive	General-purpose summarization across varied domains (e.g., internal reports)
Phi	Highest readability; lowest emotional intensity; consistent output	Least lexical diversity; may oversimplify nuanced content	Public-facing summaries requiring clarity and accessibility (e.g., public health alerts)

## 4 Discussion

### 4.1 Summary

This study highlights the potential of lightweight LLMs to support accessible public health communication through a framework called HEAL-Summ. By analyzing longitudinal summaries of health-related news across multiple dimensions such as semantic consistency, readability, lexical diversity, and emotional alignment, it was demonstrated that smaller models can distill complex content into streamlined, publicly digestible formats but may exhibit distinct stylistic and tonal tendencies. While semantic similarity remained consistently high across models, notable differences emerged in how each system shaped accessibility and tone. Qwen, on average, generated the most lexically diverse outputs that may better capture nuances, but often at the cost of readability. Phi, by contrast, favored simplicity as it consistently yielded lower grade-level summaries that may be more suitable for broad public dissemination. Llama served as a middle ground, balancing moderate complexity with relatively stable accessibility. These findings suggest that model selection should be responsive to audience needs and the communicative context.

Emotionally, summaries largely mirrored the source material, especially the dominance of fear and sadness in health reporting, but variations suggest that models subtly shape emotional framing. Phi periodically softened anger and elevated expressions of happiness, especially in topics such as suicide and vaccine, potentially aligning with ethical principles of minimizing harm. In contrast, Qwen sometimes diverged from the emotional tone of the source, which may be harmful as it fails to preserve urgency or authenticity. Such emotional variation underscores the importance of selecting and configuring LLMs with care, especially when dealing with topics that affect public perception and mental health.

While many LLMs are capable of producing summaries, HEAL-Summ distinguishes itself by offering an integrated, reproducible framework for evaluating the accessibility, emotional tone, and ethical quality of health-related news summarization across time. Rather than treating summarization as a one-off task, HEAL-Summ facilitates longitudinal tracking of public health narratives using locally deployable models. This approach is particularly valuable in settings where commercial APIs may be cost-prohibitive or unavailable, and where ethical transparency and content traceability are essential. By combining lightweight deployment with multi-dimensional evaluation, the framework bridges a crucial gap between technical feasibility and responsible real-world application.

However, this approach is not without limitations. While the reliance on publicly available news data enables transparency, it constrains the scope of the analysis as it excludes emerging health data that may only be available through private or academic sources. This introduces representational biases as news outlets may reflect perspectives that differ across regions, communities, or media types. To address this, future work should explore broader use of the MediaCloud API, incorporating more diverse sources. Such an expansion would allow for more inclusive modeling of public health narratives and a deeper analysis of representational faireness in generated summaries. Additionally, while lightweight LLMs offer efficiency, their performance may not fully match the depth and nuance achievable by larger models. The reliance on automated metrics for harmful content, consistency, and emotion analysis, though effective, may also require periodic human review to ensure contextual appropriateness. Therefore, future work should focus on evaluating additional LLMs as well as performing manual verifications with domain experts.

While this study focused exclusively on text-based inputs, HEAL-Summ could be extended to support multimodal data in future iterations. For example, health-related content embedded in videos or text-containing images (e.g., infographics or social media screenshots) could be processed using OCR and audio/visual transcription models. This would broaden the applicability of the framework to sources that contain several forms of media, and enable more comprehensive coverage of public health narratives. Lastly, while HEAL-Summ includes tools for identifying ethical concerns such as hallucination or harmful framing, these indicators are automated. The use of semantic divergence, toxicity, and emotional variation serves as a proxy for deeper content analysis, but future work should incorporate expert human review to confirm the presence of factual inconsistencies or ethical risk. Nonetheless, this strategy offers a practical and scalable alternative in high-volume or time-sensitive environments.

## 5 Conclusion

This work presents HEAL-Summ, a practical framework for generating accessible health-related summaries using lightweight LLMs. By applying the pipeline across public news articles, its flexibility and value for real-time health communication was demonstrated, especially in low-resource or time-sensitive contexts. The evaluation framework, spanning semantic alignment, readability, lexical diversity, and emotional tone, offers a multifaceted approach to assess model behavior.

The results show that while all models maintain strong semantic alignment, each exhibits distinct stylistic and emotional patterns, suggesting that model selection should be tailored to both content and audience. Emotional moderation, accessibility, and lexical diversity trade-offs are especially relevant in sensitive domains such as mental health, addiction, and suicide. The observed differences between models underscore the importance of multi-model evaluations and transparent reporting in LLM-assisted communication systems. As the framework evolves, efforts to increase source diversity and representational fairness will be essential to align LLM-driven communication with principles of public health equity.

Looking ahead, future work should prioritize participatory evaluations to validate how different audiences perceive and respond to these summaries. Incorporating domain-specific fine-tuning, multilingual expansion, and integration with community-driven health platforms will further enhance cultural relevance and reach. By making complex information not only available but understandable and trustworthy, this framework contributes to the growing effort to align LLMs with public health equity and information accessibility.

## Data Availability

The datasets presented in this study can be found in online repositories. The names of the repository/repositories and accession number(s) can be found in the article/Supplementary material.

## Footnotes

^1^ https://help.openai.com/en/articles/7127956-how-much-does-gpt-4-cost

^2^ https://github.com/aman2656/text2emotion-library

^3^ https://github.com/andrfish/FiM-Lightweight-LLM-Summarization-Framework
