# Supplementary material for: HEAL-Summ: a lightweight and ethical framework for accessible summarization of health information
Source: Front Public Health. 2025 Sep 25;13:1619274. doi: 10.3389/fpubh.2025.1619274 (PMC12507851; doi:10.3389/fpubh.2025.1619274)
Supplement: Supplementary file 1 [file Data_Sheet_1.pdf]

Supplementary Material

1 SAMPLES OF READABILITY SCORE RESULTS

Qwen Summary with Low Readability Scores (average readability score of 12.738)

The Public Health Agency of Canada has stated that it is not providing Novavax's COVID-19 vaccine this respiratory virus season due to low demand. The agency notes that Novavax requires a minimum order of its updated protein-based vaccine, Nuvalox, which is significantly higher than the uptake last year. Both the original and updated versions of the COVID-19 vaccines have been reformulated to target the KP.2 and JN.1 subvariants of Omicron. While provinces and territories can still order the vaccine directly from the manufacturer, there has been low demand for it in previous years. Additionally, some Canadians who are immunocompromised and have experienced adverse reactions to mRNA vaccines are calling the decision to stop providing Novavax unfair. Meanwhile, the agency has also indicated that provinces can order Novavax vaccines made in India. Despite these efforts, the agency emphasizes that provinces and territories have the option to order the updated version of the vaccine, which targets the JN.1 subvariant of Omicron. In contrast, the Canadian government is focusing on other vaccines such as the flu shot, which is now available for all residents aged six months and older. For respiratory syncytial virus (RSV), a new monoclonal antibody, Nirsevimab, has been approved by Health Canada and will be offered to all babies experiencing their first RSV season in Ontario, Quebec, and Nunavut this fall and winter.

Phi Summary with Low Readability Scores (average readability score of 10.330)

In recent news, two prominent figures in the world of sports and royalty have been dealing with cancer. Figure skater Tessa Virtue, who won a world bronze medal in 2023, revealed that she had undergone surgery to remove an ovary and appendix in December 2022. She shared that the experience has changed her perspective on life. Tessa's mother, Bonnie, passed away from glioblastoma, a type of brain cancer, in 2018. Tessa is actively involved in raising awareness about brain and ovarian cancer.

In the royal family, Catherine, the Princess of Wales, announced that she has been diagnosed with cancer. She revealed this in a video shared on social media, stating that tests after her surgery revealed the presence of cancer. Catherine is currently undergoing early-stage preventive chemotherapy. This announcement comes just weeks after it was revealed that King Charles III is also dealing with a cancer diagnosis. The specific type of cancer has not been disclosed.

Llama Summary with Low Readability Scores (average readability score of 12.202)

There have been several concerns raised about public health in various regions across Canada. In Kingston and the surrounding area, there has been a 50% increase in apparent drug poisonings since February 24, with emergency crews responding to 17 overdoses in just 24 hours on February 6. Meanwhile, in Thunder Bay, the number of confirmed cases of syphilis is on the rise, with a 5.8-fold increase in syphilis cases in the Thunder Bay District Health Unit area since 2019. In British Columbia, toxic drugs have killed 198 people in January, adding to an unrelenting toll that has killed more than 14,000 people since a public health emergency was declared eight years ago. Additionally, there have been concerns about the rise in syphilis cases across Canada, with the country experiencing an ”alarming increase” in syphilis cases, according to Dr. Theresa Tam, the Chief Public Health Officer of Canada. Health officials are urging the public to be aware of the risks and to take steps to prevent the spread of these diseases, such as getting vaccinated against measles and syphilis.

Qwen Summary with High Readability Scores (average readability score of 19.816)

The news articles highlight various aspects related to the topic of suicide and mental health, including the dangers posed by violent online groups targeting vulnerable minors, the ongoing youth suicide crisis among First Nations communities, discussions around medically assisted dying (MAID), and the impact of delays in expanding MAID eligibility to include mental illness. The CBC article from February 7th discusses the alarming activities of violent online groups that exploit and coerce minors into sharing harmful content, emphasizing the urgent need for intervention. Another CBC piece from February 8th addresses the persistent youth suicide crisis within First Nations communities, highlighting the lack of adequate mental health support and the need for meaningful engagement in decision-making processes. Meanwhile, the Winnipeg Free Press articles from February 6th and 8th touch upon the controversy surrounding the expansion of MAID to include mental illness, with concerns raised by medical professionals about the potential risks and ethical considerations. These pieces collectively underscore the complex and multifaceted nature of addressing suicide and mental health issues, emphasizing the importance of comprehensive support systems and timely policy decisions.

Phi Summary with High Readability Scores (average readability score of 18.594)

The news posts from CBC and Global News highlight various issues related to addiction, including the challenges faced by communities in northeast Saskatchewan following a mass stabbing event, the introduction of a drug decriminalization pilot in British Columbia, and the growing concern over the safety and accessibility of emergency shelters in Edmonton. The posts also discuss the impact of the opioid crisis in Nova Scotia, the need for safer opioid supply treatment, and the mental health toll of the COVID-19 pandemic, particularly among younger populations. Additionally, the posts mention the launch of a new provincial drug alert system in Saskatchewan and the Downtown Safety Leadership Panel’s recommendations for improving safety in Calgary’s downtown core.

Llama Summary with High Readability Scores (average readability score of 19.564)

Public health officials in Prince Edward Island are investigating an outbreak of gastrointestinal illness reported among dozens of people who attended a shellfish festival over the weekend, with over 550 people experiencing symptoms of norovirus after the event. In British Columbia, the Green Party has proposed a three-point plan to address deaths from toxic, illicit drugs, including expanding access to prescribed alternatives, improving drug education in schools, and ensuring adequate mental-health supports. Meanwhile, Ontario's health system is grappling with high inflation, rapid population growth, and complex health needs, with experts calling for a more integrated approach to public health care that complements universal public health services. Additionally, public health officials are warning of the potential for insect-borne viruses and diseases to become more commonplace in Canada due to record-breaking heat waves, with over 800 risk areas for Lyme disease identified across the country.

2 SAMPLES OF LEXICAL DIVERSITY ANALYSIS RESULTS

Qwen Summary with Low Lexical Diversity (MLTD score of 57.864)

The news articles reviewed touch upon various aspects of mental health, including the need for improved mental health support for RCMP officers, concerns over the diversion of pharmaceutical alternatives for drug users, recommendations for enhancing mental health services in Quebec, and the opioid crisis in British Columbia. Additionally, there are discussions about the adequacy of mental health resources and the need for specialized tribunals to handle mental health cases. The articles also highlight the challenges faced by individuals with mental health issues, such as the stigma surrounding mental health and the difficulties in accessing necessary support. Furthermore, there are mentions of efforts to improve mental health care, including the expansion of psychiatric residency training programs and the regulation of mental health workers. Lastly, the news includes updates on the opioid crisis in British Columbia and the potential for increased funding for mental health facilities in Toronto.

Phi Summary with Low Lexical Diversity (MLTD score of 62.258)

In recent news, mental health has been a focal point in various incidents and policy discussions across Canada. A mental health call in Peterborough led to a school being placed on hold and secure, highlighting the impact of mental health crises on community safety. A study revealing the extreme stress and low-paying jobs faced by working migrant and immigrant women underscores the need for better mental health access and support. Toronto's expansion of the emergency service for mental health crisis response, the city's partnership with local agencies to assist Calgarians in crisis, and the federal government's acknowledgment of the importance of the safe supply program in preventing overdose deaths all reflect the growing recognition of mental health issues. Additionally, concerns about the diversion of safe supply opioids in British Columbia and the need for improved mental health care for children in the child-welfare system in Ontario further illustrate the ongoing challenges and efforts to address mental health in Canada.

Llama Summary with Low Lexical Diversity (MLTD score of 66.384)

In recent weeks, several provinces in Canada have announced initiatives to improve cancer care and treatment. In Saskatchewan, the provincial government has launched several breast cancer care and screening initiatives, including a new breast health centre in Regina and the expansion of provincial breast cancer screening eligibility. In British Columbia, the government has announced a three-year, $270-million plan to expand access to new forms of cancer treatment, including immunotherapy. Additionally, Surrey Memorial Hospital in British Columbia has announced plans to hire hundreds of new healthcare workers, including doctors and nurses, to strengthen cancer care in the region. Meanwhile, a new law has been passed in the US Senate to compensate Americans exposed to radiation by the government, and a non-profit group has been launched in Canada to address the ongoing impact of COVID-19 on chronic illness, including cancer.

Qwen Summary with High Lexical Diversity (MLTD score of 334.951)

Several articles touch upon themes of addiction and its management. In Shamattawa First Nation, a band council resolution was passed to allow local and hired security officers to conduct searches, potentially targeting drugs and alcohol. This reflects broader societal concerns about substance abuse. Tiffany Halliday, a survivor of sexual exploitation, discusses her journey with the Blue Door Program at Thrive, emphasizing the distinction between sex work and sexual exploitation. Meanwhile, Jennifer Cooper from BC Health Care Cost Containment System highlights the issue of diverted safe-supply drugs from B.C., indicating organized crime groups are involved in redistributing these drugs, which can be resold outside B.C. Additionally, Brenda Doherty's story underscores the challenges faced by families dealing with drug experimentation, suggesting that legislation aimed at deterring teens from asking for help might have been beneficial. The BC Centre on Substance Use has expressed concern about the presence of confirmed safer supply prescriptions in drug seizures, noting that while there have been notable quantities seized, there is currently no evidence of widespread diversion. Lastly, the Public Health Accountability and Cost Recovery Act introduced by British Columbia aims to hold companies accountable for promoting, marketing, and distributing harmful products, including those that create addiction, particularly in children and young adults. This legislation seeks to address the significant public health harms caused by such products.

Phi Summary with High Lexical Diversity (MLTD score of 266.256)

In recent news, the public health sector has been under scrutiny due to concerns surrounding the security of Canada's only biosecurity level 4 lab in Winnipeg. A parliamentary committee has questioned key witnesses about the lab's operations, particularly the covert collaboration with China. The committee's concerns stem from a past incident where two scientists, Qiu and Cheng, were dismissed after working with China, raising fears of potential national security breaches. The government has since tightened security measures, including screening staff and visitors, to prevent similar occurrences.

Meanwhile, Canada's Chief Public Health Officer, Dr. Theresa Tam, has reported a significant increase in measles cases, more than three times higher than the previous year. Dr. Tam urges Canadians to ensure their vaccinations are up to date, especially before the busy March break travel season. The public health agency of Canada is aware of 40 confirmed cases across the country in 2024.

In addition, the issue of foreign interference in Canadian elections has been brought to light, with the hearings delving into allegations against China, India, and Russia. Canada's Chief Electoral Officer, Stéphane Perrault, is set to testify at a commission of inquiry into these allegations.

Lastly, the public health sector is also facing challenges related to staffing agencies. Hospitals and long-term care homes have reported spending nearly $1 billion last year to fill shifts with nurses and personal support workers from private staffing agencies. These agencies charge double or even triple the regular hourly rate, leading to concerns about the affordability crisis and its impact on substance use in Canada.

Llama Summary with High Lexical Diversity (MLTD score of 243.088)

The Manitoba government has been addressing concerns over mental health, with Advocate for Children and Youth Sherry Gott releasing her annual report card, which highlighted the need for improvement in mental health services. The government has also boosted funding to $800,000 for youth support programs, including the Indigenous Youth Mentorship Program, which helps university and high school students develop leadership skills. However, some policies have raised concerns, such as Alberta Premier Danielle Smith's recently announced policies regarding transgender youth, which prohibit puberty blockers and hormone therapy for those 15 and under, unless they've already started treatment. This decision has been met with criticism, with some arguing that it's forcing young people to undergo a traumatic puberty. Additionally, there have been incidents of police use of force against individuals dealing with mental health issues, including a recent inquiry launched by British Columbia's Human Rights Commissioner, Kasari Govender, which found a "disturbing pattern of discrimination in policing in the province." Furthermore, a former teacher's aide from Durham Region, Ashley Jansen, is speaking out about the need for accountability after a judge ruled that she was falsely accused of sexual assault.

3 SAMPLES OF SEMANTIC SIMILARITY ANALYSIS RESULTS ACROSS THE SAME ARTICLES

Qwen Summary with High Similarity (average similarity to other models of 0.915)

Several recent news items highlight ongoing concerns and updates regarding vaccines. Health Canada has advised provinces to remove existing supplies of the COVID-19 vaccine targeting the XBB form of Omicron, with updated vaccines targeting the JN.1 or KP.2 strains expected to be authorized in the coming weeks. Meanwhile, amid a rise in measles cases globally and a handful of confirmed cases in Canada, the National Public Health Agency strongly advises everyone to ensure they are fully immunized against measles, especially before traveling. Additionally, Ontario's Chief Medical Officer, Dr. Kieran Moore, expressed concern over a dramatic rise in global measles cases, noting that there are currently four measles cases in Canada, including two in Ontario. The situation underscores the importance of maintaining high vaccination rates to prevent outbreaks. Furthermore, Nova Scotia's health authority announced plans to lift a requirement for its employees and on-site hospital staff to submit proof of COVID-19 immunization, though this does not affect health-care workers who are already mandated to be vaccinated. These developments underscore the ongoing need for vigilance and adherence to vaccination guidelines to protect public health.

Phi Summary with High Similarity (average similarity to other models of 0.890)

In recent news, Health Canada has instructed provinces to withdraw existing supplies of the COVID-19 vaccine targeting the XBB form of Omicron, as per a spokesperson for Ontario's health minister. Updated vaccines targeting the JN.1 or KP.2 strains are expected to be authorized in the coming weeks. The U.S. FDA approved Pfizer's updated COVID-19 vaccine for individuals aged 12 and up on August 22. Health Canada is currently reviewing submissions from Pfizer, Moderna, and Novavax for updated COVID-19 vaccines for planned fall campaigns.

Meanwhile, the national public health agency is strongly advising everyone to check that they are fully immunized against measles, especially before traveling. This comes amid a rise in measles cases globally and a few confirmed cases in Canada. Public health agencies, including Toronto Public Health, are offering "catch-up" clinics for measles shots and other routine immunizations for children. Adults seeking measles vaccine without a primary care provider can find it at many walk-in clinics or travel medicine clinics.

In addition, the Globe and Mail reported that the fall COVID-19 vaccine cut the risk of COVID-19 illness by half overall. The Canadian Security Intelligence Service urged the removal of security clearances for two scientists involved in shipping exceptionally virulent viruses to the Wuhan facility. Nova Scotia's health authority announced that it is lifting the requirement for its employees and on-site hospital staff to submit proof of COVID-19 immunization, effective Monday. The government could not release documents about their dismissal for national-security reasons.

Lastly, the government has not released documents about the dismissal of two scientists for national-security reasons. Dr. Qiu, who headed the vaccine development and antiviral therapies section at the lab, and Mr. Chen were finally dismissed in January 2021. Dr. Qiu won a Governor-General's Award in 2018 for her work on Ebola.

Llama Summary with High Similarity (average similarity to other models of 0.915)

Health Canada has instructed provinces to pull existing supplies of COVID-19 vaccines targeting the XBB form of Omicron, while updated vaccines targeting the JN.1 or KP.2 strains are expected to be authorized in the coming weeks. Meanwhile, a rise in measles cases globally has prompted Health Canada to strongly advise everyone to check their immunization status, especially before traveling. The country has seen four measles cases, including two in Ontario, linked to international travel. In other news, a pharmacist has stated that the influenza vaccine was 63% effective against the dominant strain this season, and a Canadian health official has warned of the potential surge in imported measles cases, especially with spring break around the corner. Additionally, Nova Scotia's health authority has lifted a requirement for employees to submit proof of COVID-19 immunization, while a memo from Ontario's chief medical officer has highlighted the need for health system partners to be prepared for continued importation of measles cases and potential outbreaks.

Qwen Summary with Low Similarity (average similarity to other models of 0.520)

A mother from Weyburn, Saskatchewan, has been diagnosed with breast cancer, requiring urgent medical attention and a referral to Calgary for a breast biopsy. The situation highlights the importance of timely diagnosis and treatment for breast cancer patients. Additionally, King Charles III of the United Kingdom has been diagnosed with cancer, prompting a public outpouring of well-wishes and support from Canadians and around the world. This revelation comes amidst ongoing discussions about the British monarchy and its relationship with the public, particularly given the prevalence of cancer in the country- 3 million people live with cancer, according to Macmillan Cancer Support. King Charles III's diagnosis has also led to a temporary postponement of some of his public-facing duties, emphasizing the impact of such health issues on royal engagements.

Phi Summary with Low Similarity (average similarity to other models of 0.405)

In recent news, a mother from Saskatchewan has been battling breast cancer since early December, with her case being deemed urgent by a doctor who referred her to Calgary for a biopsy. Meanwhile, the Canadian rock band Econoline Crush, known for their highways tours since the early '90s, has seen its lead singer, Trevor Hurst, transition from music to nursing. Hurst took a leave to care for his newborn daughter in 2019 but returned to nursing last year. Hurst's journey from singer to nurse was not without its challenges, but he persevered, even bringing his clients to a music festival to celebrate his return. In other health news, a mother of two from Weyburn, Saskatchewan, has been diagnosed with breast cancer, and she is still waiting for a surgeon consultation five months after discovering a lump.

Llama Summary with Low Similarity (average similarity to other models of 0.635)

Several news stories have reported on the diagnosis of King Charles III with cancer, with Buckingham Palace confirming the news and Prime Minister Justin Trudeau expressing his well-wishes for the monarch's recovery. In a separate development, a mother from Saskatchewan has been diagnosed with breast cancer, highlighting the ongoing issue of breast cancer in Canada. Additionally, a Canadian rock band, Econoline Crush, has a lead singer who has overcome his own personal demons to become a nurse, and a friend encouraged him to apply for the job. Meanwhile, a Canadian Press report noted that Nova Scotia health services are still reduced following a snowstorm, and a doctor in Nova Scotia expressed concerns about the impact of the reduced services on patients. Furthermore, a Globe and Mail article discussed the challenges faced by family doctors in Canada, including burnout and long wait times to see specialists.

4 STATISTICAL SIGNIFICANCE TESTS: LEXICAL DIVERSITY AND READABILITY

Table S1. Pairwise adjusted p-values for MTLD.

Model A	Model B	Adjusted p-value
Llama	Phi	0.002
Qwen	Llama	0.000
Qwen	Phi	0.000

Table S2. Pairwise adjusted p-values for Flesch-Kincaid Grade Level (FK).

Model A	Model B	Adjusted p-value
Qwen	Phi	0.000
Qwen	Llama	0.095
Phi	Llama	0.000

Table S3. Pairwise adjusted p-values for Automated Readability Index (ARI).

Model A	Model B	Adjusted p-value
Qwen	Phi	0.000
Qwen	Llama	0.001
Phi	Llama	0.000

Table S4. Pairwise adjusted p-values for Coleman-Liau Index (CLI).

Model A	Model B	Adjusted p-value
Qwen	Phi	0.000
Qwen	Llama	0.000
Phi	Llama	0.000

Table S5. Pairwise adjusted p-values for Dale-Chall Readability Score (DC).

Model A	Model B	Adjusted p-value
Qwen	Phi	0.000
Qwen	Llama	0.003
Phi	Llama	0.000

Table S6. Pairwise adjusted p-values for SMOG Index.

Model A	Model B	Adjusted p-value
Qwen	Phi	0.000
Qwen	Llama	0.005
Phi	Llama	0.000

5 STATISTICAL SIGNIFICANCE TESTS: EMOTIONS

Table S7. Pairwise adjusted p-values for the ”Fear” emotion score across models.

Model A	Model B	Adjusted p-value
Qwen	Phi	0.000
Qwen	Llama	0.000
Phi	Llama	1.000

Table S8. Pairwise adjusted p-values for the “Angry” emotion score across models.

Model A	Model B	Adjusted p-value
Qwen	Phi	0.000
Qwen	Llama	0.000
Phi	Llama	0.016

Table S9. Pairwise adjusted p-values for the “Happy” emotion score across models.

Model A	Model B	Adjusted p-value
Qwen	Phi	0.000
Qwen	Llama	0.001
Phi	Llama	0.122

Table S10. Pairwise adjusted p-values for the “Sad” emotion score across models.

Model A	Model B	Adjusted p-value
Qwen	Phi	1.000
Qwen	Llama	0.000
Phi	Llama	0.001

Table S11. Pairwise adjusted p-values for the “Surprise” emotion score across models.

Model A	Model B	Adjusted p-value
Qwen	Phi	0.000
Qwen	Llama	0.000
Phi	Llama	1.000
